# Mortality attributed to sickle cell disease in children and adolescents in Brazil, 2000–2019

**DOI:** 10.11606/s1518-8787.2022056003681

**Published:** 2022-06-27

**Authors:** Maria Isabel do Nascimento, Ana Luísa Ferreira Przibilski, Carolina Sampaio Gomes Coelho, Katyslaine Frossard de Amorim Leite, Mariana Makenze, Stella Bayer de Jesus

**Affiliations:** I Universidade Federal Fluminense Faculdade de Medicina Niterói RJ Brasil Universidade Federal Fluminense. Faculdade de Medicina. Mestrado Profissional em Saúde Materno Infantil. Niterói, RJ, Brasil; II Universidade Federal Fluminense Faculdade de Medicina Programa de Iniciação Científica Niterói RJ Brasil Universidade Federal Fluminense. Faculdade de Medicina. Programa de Iniciação Científica. Niterói, RJ, Brasil

**Keywords:** Child, Adolescent, Anemia, Sickle Cell, epidemiology, Mortality, trends, Time Series Studies

## Abstract

**OBJECTIVE:**

Estimate rates and describe mortality trends attributed to sickle cell disease in children and adolescents in Brazil from 2000 to 2019.

**METHODS:**

This is an ecological study of the time-trend of mortality rates that used the autoregressive method, proposed by Prais-Winsten, to evaluate trends in the estimated rates of sickle cell disease deaths in children and adolescents in Brazil. Deaths with code D57 were obtained from the Mortality Information System, considering age groups (0–4, 5–9, 10–14, 15–19 years) and were used to estimate age-specific and standardized rates by gender and age.

**RESULTS:**

From 2000 to 2019, Brazil had 2,422 deaths from sickle cell disease in people under 20 years of age, with higher frequency in the Northeast (40.46%), followed by the Southeast (39.02%), Midwest (9.58%), North (7.84%), and South (3.10%). The main victims were people of Black skin/race (78.73%). In Brazil, the global standardized average rate was 0.20/100,000 people-year, with an elevation trend (annual percentage change – APC = 5.44%; confidence interval – 95%CI: 2.57–8.39). The pattern was repeated in males (APC = 4.38%; 95%CI: 2.17–6.64) and females (APC = 6.96%; 95%CI: 3.05–11.01). Elaborating age-specific rates showed that the range up to four years experienced the highest rates, without distinction by region. The age group of 15 and 19 years was the second most affected in Brazil and in the Northeast, Southeast, and Midwest regions.

**CONCLUSION:**

Deaths due to sickle cell disorders showed an elevation trend in children and adolescents. Considering that the magnitude of deaths was more evident in the first years (0–4) and late adolescence (15–19), the study suggests that age-specific approaches may impact the control of fatal outcomes caused by sickle cell disease in Brazil.

## INTRODUCTION

Sickle cell disease (SCD) encompasses a group of β-hemoglobinopathies characterized by the predominance of sickle cell hemoglobin (HbS) within erythrocytes. The HbS mutation makes hemoglobin more likely to polymerize and damage the erythrocyte membrane, which assumes the appearance of a sickle. The useful life of these erythrocytes is reduced, culminating in hemolytic anemia, with painful vaso-occlusive manifestations, ischemic endothelial dysfunction and chronic inflammatory response^1^.

Hemoglobinopathies are the most frequent monogenic genetic diseases in the world, affecting approximately 7% of the world population^2^. Global estimates showed that, in 2010, 5,476,407 and 312,302 newborn babies could be affected by genetic defects of heterozygous hemoglobin (AS) and homozygous hemoglobin (SS), respectively^3^. In Brazil, 3.7% of the adult population reported hemoglobinopathies, and the sickle cell trait (2.49%) and thalassemia minor (0.8%) were the most prevalent types^4^. The Ministry of Health reports the incidence of sickle cell trait in 1:35 live births and estimates that, annually, 3,000 children are born with SCD and another 200,000 presenting traces of the disease^5^.

The year 2001 marked the inclusion of SCD screening in the National Neonatal Screening Program (PNTN) in the country and created an opportune scenario for the early implementation of clinical approaches aimed at preventing complications^6^. Recently, a review of international studies found scarce publications addressing the monitoring of this genetic anomaly by mortality studies^7^. A synthesis of seven studies conducted in Brazil showed that the indicators were only restricted to some federative units^8^, suggesting that studies focusing on SCD that make evident the magnitude of deaths on a national scale are lacking, particularly in the children and adolescents’ segment, after the implementation of screening by the PNTN. Thus, this study aimed to describe the mortality rates attributed to sickle cell disease in children and adolescents, in Brazil and in its geographic regions, from 2000 to 2019.

## METHODS

This is an ecological time series study that used an autoregressive modeling to describe the strength and trends with which SCD caused deaths in children and adolescents in Brazil, from the year 2000. The data were collected on the website of the *Departamento de Informática do Sistema Único de Saúde* (Datasus – Department of Informatics of the Unified Health System)^9^, consulting health information and vital statistics. The population data were extracted from the database of the *Instituto Brasileiro de Geografia e Estatística* (IBGE – Brazilian Institute of Geography and Statistics)^10^.

### Variables of Interest

The study covered Brazil as a whole and the five geographic regions separately (North, Northeast, Southeast, South, and Midwest) and focused on deaths with underlying cause of SCD (D57), according to the International Statistical Classification of Diseases and Related Health Problems – ICD 10. The research period covered the years 2000 to 2019, which comprised 10 biennia, starting in 2000–2001 and ending in 2018–2019. Correlation analyses between rates and biennia included gender (male and female) and age group (0–4, 5–9, 10–14, 15–19 years), as specified on the Datasus website.

Anticipating the importance of the race/skin color profile for sickle cell outcomes, the study provided a brief description of the distribution of deaths regarding this marker, in addition to age and gender. The race/skin color item was described according to the strata (white, black, mixed race, yellow, and indigenous) used in the TABNET platform of Datasus^9^ and the category “black”, corresponds to the sum of “mixed race” and “black”, as used by IBGE^10^.

### Mortality Rates

To calculate mortality rates, the number of deaths from Datasus was used as numerator and the population contingent projected by IBGE as denominator. The age-standardized mortality rates were calculated for each of the 10 biennia, considering the four age groups under analysis and the total Brazilian population targeted in the study. The rates were standardized by the direct method with reference to the world population^11^. Age-specific mortality rates were calculated for the five macro-regions and to Brazil as a whole, considering the four age groups. All indicators were presented per 100,000 people-year.

### Time Trends

The indicators were arranged in line charts with the dependent variable (rate) on the *y* axis and the independent variable (biennium) on the *x* axis. The age-adjusted rates were calculated only for Brazil and were arranged considering the total Brazilian population targeted by the study and the strata defined by gender. Age-specific rates were plotted according to age groups, after smoothing the coefficients, using the technique of third order moving averages, and presented with results from Brazil and the five macro-regions.

### Statistical Analysis

The statistical analysis was conducted using linear regression to verify the behavior of the estimated adjusted rates for Brazil. Seeking to reduce the effect of residual autocorrelation, determined by the proximity of the events computed throughout the biennia, the study followed the recommendations of Antunes and Cardoso^12^ and applied the autoregressive method of Prais-Winsten^13^. The annual percentage changes (APC) were calculated after logarithmic transformation of the coefficients and presented with their respective 95% confidence intervals (95%CI). Trends were interpreted as stationary, decline or elevation. The analyses were conducted using the Microsoft Excel program^®^ and the R platform.

### Ethical Aspects

The study followed the International Ethical Guidelines for Health-related Research Involving Humans Beings and was developed with secondary data, made publicly available, online, by Datasus and IBGE, and is therefore free of formal ethical procedures.

## RESULTS

From 2000 to 2019, Brazil had 2,422 deaths from sickle cell disease among children and adolescents. The frequency was higher in males (54.24% *versus* 45.75%) and in the age group from zero to nine years (55.20% *versus* 45.80). The distribution by macro-region showed higher frequency in the Northeast (40.46%), followed by the Southeast (39.02%), Midwest (9.58%), North (7.84%), and South (3.10%).

In view of the importance of race/skin color in SCD studies, we analyzed the lack of information in this item. We observed that the proportion of data lost was 9.54%, which represents a considerable reduction, from 21.51% in 2000 to less than 10% from 2009 (8.06%), and to 3.01% in 2019. From the set of deaths with the variable race/skin color adequately registered (n = 2,191), we observed that the majority of fatal victims are people who were classified as black, representing 78.73% of deaths in Brazil and in four of the macro-regions: 85.16% of deaths in the North, 86.71% in the Northeast, 71.16% in the Southeast, and 80.47% in the Midwest. The South region, which has the highest concentration of white population in the country, showed a distribution of deaths by skin color of 50% in both whites and blacks (Table 1).

**Table 1:** Deaths attributed to sickle cell disease in children and adolescents: distribution by age, gender, and skin color, according to Brazilian macro-regions, from 2000 to 2019.

Characteristics	Brazil	Geographic macro-regions				
		North	Northeast	Southeast	South	Midwest
	n = 2,191	n = 182	n = 869	n = 853	n = 72	n = 215
Age (years)	n (%)	n (%)	n(%)	n (%)	n (%)	n (%)
0–4	797 (36.38)	74 (40.66)	325 (37.40)	297 (34.82)	23 (31.94)	78 (36.28)
5–9	406 (18.53)	39 (21.43)	163 (18.76)	147 (17.23)	12 (16.67)	45 (20.93)
10–14	344 (15.70)	26 (14.28)	133 (15.30)	142 (16.65)	15 (20.83)	28 (13.02)
15–19	644 (29.39)	43 (23.63)	248 (28.54)	267 (31.30)	22 (30.56)	64 (29.77)
Gender						
Male	1,189 (54.3)	111 (60.99)	467 (53.73)	456 (53.5)	38 (52.8)	117 (54.4)
Female	1,002 (45.7)	71 (39.01)	402 (46.26)	397 (46.5)	34 (47.2)	98 (45.6)
Skin color/Race						
White	455 (20.77)	26 (14.29)	112 (12.89)	241 (28.25)	36 (50.00)	40 (18.60)
Black	416 (18.99)	17 (9.34)	147 (16.92)	207 (24.27)	21 (29.17)	24 (11.16)
Yellow	7 (0.32)	1 (0.55)	1 (0.11)	4 (0.47)	0 (0.00)	1 (0.47)
Mixed race	1,309 (59.74)	138 (75.82)	607 (69.85)	400 (46.89)	15 (20.83)	149 (69.30)
Indigenous	4 (0.18)	0.00	2 (0.23)	1 (0.12)	0 (0.00)	1 (0.47)

**Figure 1:**
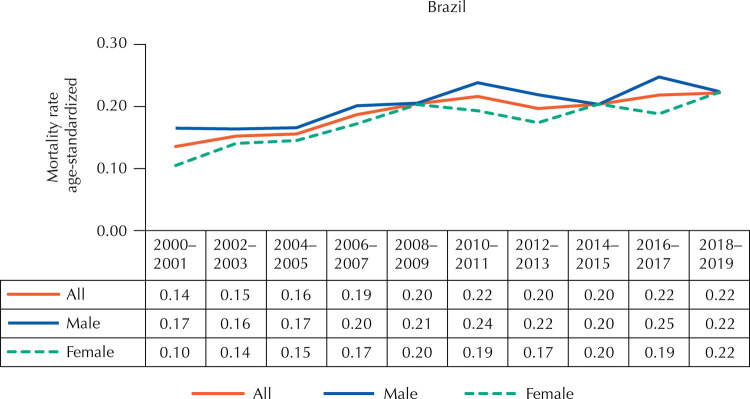
Age-standardized mortality rates per 100,000 people-year attributed to sickle cell disease in children (from zero to nine years) and adolescents (10–19 years), by bienniums, starting in 2000–2001 and ending in 2018–2019 in Brazil.

In Brazil, age-standardized mortality rates remained below 0.30 per 100,000 people-year throughout all biennia in the series. However, the rates presented a differential by gender, since the strength of the disease on deaths in the population as a whole, and in females, did not exceed that found in males in any biennium analyzed (Figure 1). The trend analysis indicated a temporal elevation of mortality rates attributed to SCD in the three population strata. In the entire population, the behavior of these deaths presented APC with a mean increase of 5.44% (95%CI: 2.57–8.39). In male children and adolescents, APC was 4.38% (95%CI: 2.17–6.64) and in females it was 6.96% (95%CI: 3.05–11.01).

The epidemiological profile expressed by age-specific rates presented a particular pattern. SCD mortality was higher in the two age range extremities analyzed. In Brazil, the population of children aged from zero to four years had rates of 0.20 and 0.32 per 100,000 people-year in the initial biennium (2000–2001) and at the end (2018–2019), respectively. In the age group from 15 to 19 years, age-specific rates were 0.13 and 0.28 per 100,000 people-year in the same biennia. Regionally, the highest magnitude of deaths was repeated in the age group from zero to four years, but the pattern changed slightly in the age groups from five to nine years and from 10 to 14 years in the North and South regions, which presented higher rates than those observed in the 15 to 19 years age group (Table 2).

**Table 2:** Age-specific rates attributed to sickle cell disease in children and adolescents, for bienniums, starting in 2000–2001 and ending in 2018–2019, in Brazil.

Location/biennium	Rates^a^ in children		Rates^a^ in adolescents	
	0–4 years	5–9 years	10–14 years	15–19 years
Brazil				
2000–2001	0.20	0.11	0.08	0.13
2018–2019	0.32	0.16	0.09	0.28
North				
2000–2001	0.22	0.08	0.03	0.03
2018–2019	0.31	0.17	0.03	0.20
Northeast				
2000–2001	0.23	0.08	0.11	0.16
2018–2019	0.45	0.17	0.16	0.51
Southeast				
2000–2001	0.20	0.16	0.07	0.15
2018–2019	0.27	0.16	0.07	0.21
South				
2000–2001	0.08	0.02	0.06	0
2018–2019	0.05	0.05	0	0.07
Midwest				
2000–2001	0.25	0.12	0.08	0.24
2018–2019	0.49	0.30	0.12	0.26

^a^ Age-specific rates presented per 100,000 people-year.

**Figure 2:**
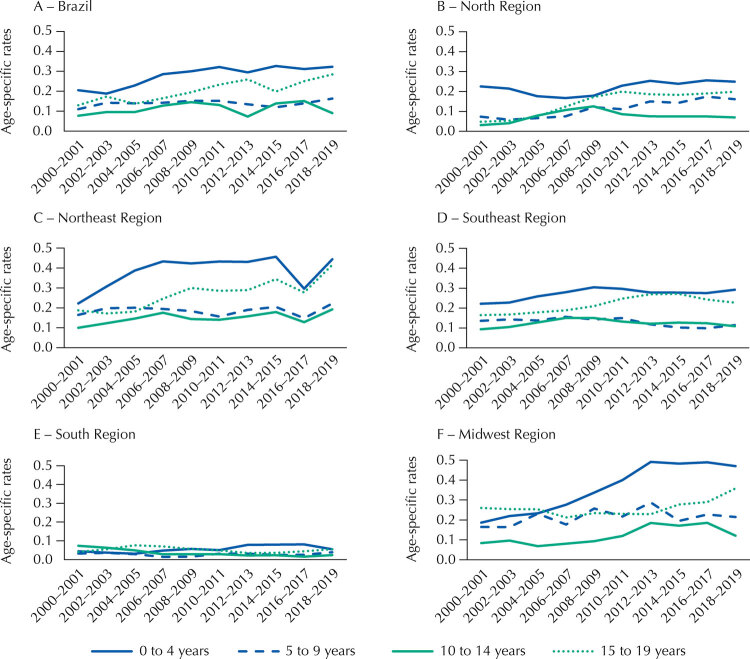
Trends in mortality rates due to sickle cell disease, age-specific in children and adolescents, per 100,000 people-year, in Brazil (a) and in the geographic macro-regions (North (b), Northeast (c), Southeast (d), South (e), Midwest (f)), by bienniums, starting in 2000–2001 and ending in 2018–2019.

Visualizing the temporal disposition of the coefficients in graphs allows the interpretation of higher magnitude of deaths in the age groups from zero to four years and from 15 to 19 years, while suggesting a trend of rates increasing over time, both in Brazil and in the macro regions (Figure 2).

## DISCUSSION

The study had the merit of analyzing sickle cell disease, a topic still scarcely explored scientifically in Brazil, despite causing important suffering to patients and their families. The focus on children and adolescents revealed that the age groups from zero to four years and from 15 to 19 years experienced the highest mortality rates, both in Brazil as a whole and in the macro-regions. Adjusted mortality rates point to a trend of these deaths increasing over the years in the country and suggest that, on average, Brazil loses about 125 children annually due to complications of sickle cell disorders.

We could verify that mixed race and black children and adolescents are the main fatal victims of SCD both in the national and the regional analysis. Despite being a common characteristic of the disease^14^, the goals of the National Policy of Integral Health of the Black Population^15^ must be kept in mind, which emphasize the need to prioritize actions aimed at reducing ethnic-racial disparities in health conditions, highlighting SCD. In the ethnic-racial context, it stands out that, even in the South region, characterized by the dominance of the white population, half of the death number was recorded in white people and the other half in black people. Considering the typical miscegenation of the Brazilian population, the proportional distribution is expected to be possibly even greater among Afro-descendants than those presented in this study.

The two most affected age groups (zero to four and 15 to 19 years) also deserve reflection, since they represent moments of life with different meaning, demands, and needs. In the first years, the expectation would be the greater use of the National Policy for Comprehensive Care of Persons with Sickle Cell Disease and Other Hemoglobinopathies^16^, which guarantees the follow-up of people diagnosed by the National Neonatal Screening Program, offering effective therapeutic approaches, expanded educational measures to family members, increased access to specialized health services and, consequently, reduction of morbidity and mortality in children born with sickle cell defects^16^. But the stratum of zero to four years is precisely the one that experiences the highest death rates indistinctly in Brazil and in the geographical regions.

An evaluation of causes of death in people with SCD in Brazil showed that 10.4% of deaths occurred in children under five years of age^17^. In this study, the higher rates and an elevation trend of the stratum 0–4 years suggests that investments in SCD made so far lack greater effectiveness. Identifying where the failures are occurring is necessary to propose improvement measures that range from the adoption and/or revision of protocols to the expansion of these children’s access to health services that can make a difference in health conditions, quality of life, and survival with the disease.

The mortality of the disease is also important over the age group between 15 and 19 years, which is the second stratum in number of deaths, outlining a scenario of losses among those who overcame a long history of crises, illnesses, and hospitalizations. This issue needs to be investigated to find out what could be happening in late adolescence, causing an increase in the number of deaths due to SCD, a genetic defect that was controlled during the first years of life. Elucidating this problem will be essential to realign attention in this phase of life, which is full of challenges and uncertainties, aggravated by SCD. On the practical side, maintaining the treatment of the therapeutic regimen is a great challenge that the health professional needs to work on^18^. On the scientific side, filling gaps is necessary with the production of high-level evidence, clarifying, for example, the impact of the change in the place of care, which is now offered in environments intended for adults^19^.

In Brazil, the rate of deaths in the < 5 age group increased by 60% comparing the 2000–2001 and 2018–2019 biennia. A study restricted to the black population in the United States pointed to the decline in deaths in the same age group with rates falling from 2.05 (1979–1989) to 0.47 (2015–2017) per 100,000 people-year^20^. In France, a 15-year follow-up study^21^, which ended in 2015, investigated the cause of death in children diagnosed with SCD at birth. The authors showed that complications involving infection and anemia were the most frequent causes and more than half of the deaths shared both conditions.

SCD has been gaining attention from Brazilian authorities since the beginning of the 21st century, even if slowly, with the creation of the National Neonatal Screening Program^6^, in which some responsibilities and attributions were defined. The National Policy for Comprehensive Care of Persons with Sickle Cell Disease and Other Hemoglobinopathies was launched in 2005 and emphasized the approach to the problem in the Unified Health System^16^. General measures were addressed in the Health Education Manual of the Ministry of Health^18^. In 2009, the disease was listed among the priorities defined by the National Comprehensive Health Policy for the Black Population^15^. A clinical protocol document and therapeutic guidelines were prepared in 2010, in which the use of hydroxyurea was evaluated^22^. The decision to incorporate hydroxyurea with an appropriate therapeutic dose for children aged two years or older was published in 2013^23^. The basic conducts for the treatment of SCD, launched in 2012, emphasize the adequacy of prophylactic measures, especially in children^5^. Despite all this framework of public policies, mortality in children by SCD is still increasing in the country. Infections, acute thoracic syndrome, and acute splenic sequestering are preventable but commonly reported conditions^8^, demonstrating that attention is still needed to discover the flaws and fix them.

The SCD penalizes patients doubly, considering both the avoidability of clinical manifestations and the low visibility that it experiences in Brazil, despite constituting a public health problem in developing countries^24^. In this sense, this study had the merit of addressing mortality indicators at the national level, however, we should list some limitations. First, considering the importance of epidemiological studies and their contribution to health policies aimed at reducing racial inequalities in health, one limitation was not estimating rates according to skin color, due to the unavailability of stratified population data according to this characteristic for each age group and time frame. However, we synthesized the demographic distribution, which reinforced the importance of the problem in the mixed race and black population group, regionally or nationally.

Another limitation was the focus on deaths coded as D57, not covering underlying causes attributed to other hemoglobinopathies (D56). Although the possibility of error in the classification of deaths and underestimation of rates should not be ignored, our results are close to those reported for children in the state of Maranhão^25^.

## CONCLUSION

The study showed that deaths due to sickle cell disorders had an elevation trend in children and adolescents. Considering that the magnitude of deaths was more evident in the first years (0–4) and late adolescence (15–19), the study suggests that age-specific approaches may impact the control of fatal outcomes caused by SCD in Brazil.
