# Supplementary material for: Mortality attributed to sickle cell disease in children and adolescents in Brazil, 2000–2019
Source: Rev Saude Publica. 2022 Jun 27;56:65. doi: 10.11606/s1518-8787.2022056003681 (PMC9239544; doi:10.11606/s1518-8787.2022056003681)
Supplement: Translation

# Translation: Mortalidade atribuída à doença falciforme em crianças e adolescentes no Brasil, 2000–2019

**Authors:** Maria Isabel do Nascimento, Ana Luísa Ferreira Przibilski, Carolina Sampaio Gomes Coelho, Katyslaine Frossard de Amorim Leite, Mariana Makenze, Stella Bayer de Jesus

**Affiliations:** I Brasil Universidade Federal Fluminense. Faculdade de Medicina. Mestrado Profissional em Saúde Materno Infantil. Niterói, RJ, Brasil; II Brasil Universidade Federal Fluminense. Faculdade de Medicina. Programa de Iniciação Científica. Niterói, RJ, Brasil

**Keywords:** Criança, Adolescente, Anemia Falciforme, epidemiologia, Mortalidade, tendências, Estudos de Séries Temporais

## Abstract

**OBJETIVO:**

Estimar taxas e descrever tendências de mortalidade atribuídas à doença falciforme em crianças e adolescentes no Brasil, de 2000 a 2019.

**MÉTODOS:**

Este é um estudo ecológico do tipo séries temporais de taxas de mortalidade que usou o método autorregressivo, proposto por Prais-Winsten, para avaliar tendências das taxas estimadas de mortes por doença falciforme em crianças e adolescentes no Brasil. Os óbitos com código D57 foram obtidos no Sistema de Informações sobre Mortalidade, considerando as faixas etárias (0–4, 5–9, 10–14, 15–19 anos) e usados para estimar taxas específicas por idade e taxas padronizadas por sexo e idade.

**RESULTADOS:**

De 2000 a 2019, houve 2.422 óbitos por doença falciforme em menores de 20 anos no Brasil, com maior frequência na região Nordeste (40,46%), seguida de Sudeste (39,02%), Centro-Oeste (9,58%), Norte (7,84%) e Sul (3,10%). As principais vítimas foram pessoas de raça/cor da pele negra (78,73%). No Brasil, a taxa média padronizada global foi de 0,20/100 mil pessoa-ano, com tendência de elevação (mudança percentual anual – APC = 5,44%; intervalo de confiança – IC95% 2,57–8,39). O padrão se repetiu no sexo masculino (APC = 4,38%; IC95% 2,17–6,64) e no sexo feminino (APC = 6,96%; IC95% 3,05–11,01). A elaboração de taxas específicas por idade mostrou que a faixa até quatro anos experimentou as maiores taxas, sem distinção por região. A faixa etária de 15 e 19 anos foi a segunda mais afetada no Brasil e nas regiões Nordeste, Sudeste e Centro-Oeste.

**CONCLUSÃO:**

Houve tendência de aumento dos óbitos por transtornos falciformes em crianças e adolescentes. Considerando que a magnitude dos óbitos foi mais evidente nos primeiros anos (0–4) e no final da adolescência (15–19), o estudo sugere que abordagens específicas por faixa etária podem impactar no controle dos desfechos fatais causados pela doença falciforme no Brasil.

## INTRODUÇÃO

A doença falciforme (DF) engloba um grupo de β-hemoglobinopatias caracterizada pela predominância da hemoglobina falciforme (HbS) no interior dos eritrócitos. A mutação HbS torna a hemoglobina mais propensa a se polimerizar e danificar a membrana do eritrócito, o qual assume o aspecto de foice. A vida útil desses eritrócitos é reduzida, culminando em uma anemia hemolítica, com manifestações vaso-oclusivas dolorosas, disfunção endotelial isquêmica e resposta inflamatória crônica^1^.

As hemoglobinopatias são as doenças genéticas monogênicas mais frequentes no mundo, estando presente em aproximadamente 7% da população mundial^2^. Estimativas globais mostraram que no ano 2010, 5.476.407 e 312.302 bebês recém-nascidos poderiam estar afetados por defeitos genéticos da hemoglobina do tipo heterozigóticos (AS) e homozigóticos (SS), respectivamente^3^. No Brasil, a presença de hemoglobinopatias foi detectada em 3,7% da população adulta, sendo que o traço falciforme (2,49%) e a talassemia menor (0,8%) foram os tipos mais prevalentes^4^. O Ministério da Saúde reporta a incidência do traço falciforme em 1:35 nascidos vivos e estima que, anualmente, ocorra o nascimento de três mil crianças com DF e outras 200 mil apresentando traços da doença^5^.

O ano de 2001 marcou a inclusão do rastreamento da DF no Programa Nacional de Triagem Neonatal (PNTN) no país e criou um cenário oportuno para a implementação precoce de abordagens clínicas visando à prevenção de complicações^6^. Recentemente, uma revisão de estudos internacionais constatou a escassez de publicações abordando o monitoramento dessa anomalia genética via estudos de mortalidade^7^. Uma síntese de sete estudos conduzidos no Brasil mostrou que os indicadores apenas se restringiam à algumas unidades federativas^8^, sugerindo que há falta de estudos com enfoque na DF que tornem evidente a magnitude dos óbitos em escala nacional, particularmente no segmento de crianças e adolescentes, após a implantação da triagem pelo PNTN. Assim, o objetivo deste estudo foi descrever as taxas de mortalidade atribuída à doença falciforme em crianças e adolescentes, no Brasil e nas grandes regiões geográficas, de 2000 a 2019.

## MÉTODOS

Trata-se de um estudo ecológico do tipo séries temporais que usou uma modelagem autorregressiva para descrever a força e as tendências com que a DF provocou óbitos em crianças e adolescentes no Brasil, a partir do ano 2000. Os dados foram captados no site do Departamento de Informática do Sistema Único de Saúde (Datasus)^9^, consultando informações em saúde e estatísticas vitais. As estimativas populacionais foram obtidas no site do Instituto Brasileiro de Geografia e Estatística (IBGE)^10^.

### Variáveis de Interesse

O estudo abrangeu o Brasil como um todo e as cinco regiões geográficas separadamente (Norte, Nordeste, Sudeste, Sul e Centro Oeste) e teve enfoque nos óbitos com causa básica de DF (D57), conforme a Classificação Estatística Internacional de Doenças e Problemas Relacionados à Saúde – CID 10. O período pesquisado englobou os anos de 2000 até 2019, que compuseram 10 biênios, iniciados em 2000–2001 e finalizados em 2018–2019. As análises de correlação entre taxas e biênios abrangeram sexo (masculino e feminino) e faixa etária (0–4, 5–9, 10–14, 15–19 anos), conforme especificado no site do Datasus.

Antecipando a importância do perfil raça/cor da pele para os desfechos falciformes, o estudo forneceu uma breve descrição da distribuição dos óbitos também por esse marcador, além de idade e sexo. O quesito raça/cor foi descrito conforme os estratos (branco, preto, pardo, amarelo e indígena) utilizados na plataforma Tabnet do Datasus^9^e a categoria “negro”, corresponde à soma de “pardos” e de “pretos”, conforme utilizado pelo IBGE^10^.

### Taxas de Mortalidade

Para o cálculo das taxas de mortalidade foram utilizados, no numerador, o número de óbitos captados no Datasus e, no denominador, o contingente populacional projetado pelo IBGE. As taxas de mortalidade padronizadas por idade foram calculadas para cada um dos 10 biênios, considerando as quatro faixas etárias em análise e o conjunto da população brasileira alvo do estudo. As taxas foram padronizadas pelo método direto tendo como referência a população mundial^11^. As taxas de mortalidade específicas por idade foram calculadas para as cinco macrorregiões, além do Brasil como um todo, considerando as quatro faixas etárias. Todos os indicadores foram apresentados por 100.000 pessoas-ano.

### Tendências Temporais

Os indicadores foram dispostos em gráficos de linha tendo no eixo de *y* a variável dependente (taxa) e no eixo de *x* a variável independente (biênio). As taxas ajustadas por idade foram calculadas apenas para o Brasil e foram dispostas considerando o conjunto da população brasileira alvo do estudo, bem como estratos definidos por sexo. As taxas específicas por idade foram plotadas segundo faixas etárias, após alisamento dos coeficientes, utilizando a técnica de médias móveis de terceira ordem, sendo apresentadas com resultados do Brasil e das cinco macrorregiões.

### Análise Estatística

A análise estatística foi conduzida empregando-se a regressão linear para verificar o comportamento das taxas ajustadas estimadas para o Brasil. Buscando reduzir o efeito da autocorrelação de resíduos, determinada pela proximidade dos eventos computados ao longo dos biênios, o estudo seguiu as recomendações de Antunes e Cardoso^12^ e aplicou o método autorregressivo de Prais-Winsten^13^. As mudanças percentuais anuais (APC) foram calculadas após transformação logarítmica dos coeficientes e apresentadas com seus respectivos intervalos de confiança de 95% (IC95%). As tendências foram interpretadas como estacionária, declínio ou elevação. As análises foram conduzidas com o uso do programa Microsoft Excel^®^ e da plataforma R.

### Aspectos Éticos

O estudo obedeceu às Diretrizes Internacionais para Desenvolvimento de Pesquisa Envolvendo Seres Humanos e foi desenvolvido com dados secundários, disponibilizados publicamente, online, pelo Datasus e IBGE, estando, portanto, livre de procedimentos éticos formais.

## RESULTADOS

De 2000 a 2019, ocorreram 2.422 óbitos por doença falciforme entre crianças e adolescentes no Brasil. A frequência foi maior no sexo masculino (54,24% *versus* 45,75%) e na faixa etária de zero a nove anos (55,20% *versus* 45,80). A distribuição por macrorregião mostrou maior frequência na região Nordeste (40,46%), seguida da Sudeste (39,02%), Centro-Oeste (9,58%), Norte (7,84%) e Sul (3,10%).

Tendo em vista a importância da raça/cor da pele em estudos de DF, analisou-se a falta de informação nesse quesito. Observou-se que a proporção de dados perdidos foi de 9,54%, o que representa uma considerável redução, passando de 21,51% no ano 2000 para menos de 10% a partir de 2009 (8,06%), caindo para 3,01% em 2019. A partir do conjunto de óbitos com a variável raça/cor da pele adequadamente registrada (n = 2.191), observou-se que a maioria das vítimas fatais são pessoas que foram classificadas como negras, representando 78,73% dos óbitos no Brasil e em quatro das macrorregiões: 85,16% das mortes na Região Norte, 86,71% no Nordeste, 71,16% no Sudeste, e 80,47% no Centro-Oeste. Na região Sul, onde encontra-se a maior concentração de população branca do país, a distribuição de óbitos por cor da pele foi de 50% tanto em brancos quanto em negros (Tabela 1).

**Tabela 1:** Óbitos atribuídos à doença falciforme em crianças e adolescentes: distribuição por idade, sexo e cor da pele, segundo as Macrorregiões do Brasil, de 2000–2019.

Características	Brasil	Macrorregiões geográficas				
		Norte	Nordeste	Sudeste	Sul	Centro-Oeste
	n = 2.191	n = 182	n = 869	n = 853	n = 72	n = 215
Idade (anos)	n (%)	n (%)	n(%)	n (%)	n (%)	n (%)
0–4	797 (36,38)	74 (40,66)	325 (37,40)	297 (34,82)	23 (31,94)	78 (36,28)
5–9	406 (18,53)	39 (21,43)	163 (18,76)	147 (17,23)	12 (16,67)	45 (20,93)
10–14	344 (15,70)	26 (14,28)	133 (15,30)	142 (16,65)	15 (20,83)	28 (13,02)
15–19	644 (29,39)	43 (23,63)	248 (28,54)	267 (31,30)	22 (30,56)	64 (29,77)
Sexo						
Masculino	1.189 (54,3)	111 (60,99)	467 (53,73)	456 (53,5)	38 (52,8)	117 (54,4)
Feminino	1.002 (45,7)	71 (39,01)	402 (46,26)	397 (46,5)	34 (47,2)	98 (45,6)
Cor/raça						
Branco	455 (20,77)	26 (14,29)	112 (12,89)	241 (28,25)	36 (50,00)	40 (18,60)
Preto	416 (18,99)	17 (9,34)	147 (16,92)	207 (24,27)	21 (29,17)	24 (11,16)
Amarelo	7 (0,32)	1 (0,55)	1 (0,11)	4 (0,47)	0 (0,00)	1 (0,47)
Pardo	1.309 (59,74)	138 (75,82)	607 (69,85)	400 (46,89)	15 (20,83)	149 (69,30)
Indígena	4 (0,18)	0,00	2 (0,23)	1 (0,12)	0 (0,00)	1 (0,47)

No Brasil, as taxas de mortalidade padronizadas por idade permaneceram abaixo de 0,30 por 100 mil pessoas-ano ao longo de todos os biênios da série. No entanto, as taxas apresentaram um diferencial por sexo, pois a força da doença sobre os óbitos no conjunto da população, bem como no sexo feminino, não superou a encontrada no sexo masculino em nenhum biênio analisado (Figura 1). A análise de tendência indicou um crescimento temporal das taxas de mortalidade atribuídas à DF nos três estratos populacionais. No conjunto da população, o comportamento desses óbitos apresentou APC com elevação média de 5,44% (IC95% 2,57–8,39). Nas crianças e adolescentes do sexo masculino, o APC foi de 4,38% (IC95% 2,17–6,64) e no sexo feminino foi de 6,96% (IC95% 3,05–11,01).

**Figura 1:** Taxas de mortalidade padronizadas por idade, por 100 mil pessoas-ano, atribuídas à doença falciforme, em crianças (de zero a nove anos) e adolescentes (de 10–19 anos), por biênios, iniciando em 2000–2001 e finalizando em 2018–2019, no Brasil.

O perfil epidemiológico expressado por taxas específicas por idade apresentou um padrão particular. A mortalidade da DF foi maior nas duas faixas extremas de idade analisadas. No Brasil, a população de crianças da faixa etária de zero a quatro anos apresentou taxas de 0,20 e 0,32 por 100 mil pessoas-ano no biênio inicial (2000–2001) e no final (2018–2019), respectivamente. Na faixa etária de 15 a 19 anos as taxas específicas por idade foram 0,13 e 0,28 por 100 mil pessoas-ano, nos mesmos biênios. Regionalmente, a magnitude mais elevada de óbitos se repetiu na faixa etária de zero a quatro anos, mas mudou um pouco o padrão nas faixas etárias de cinco a nove anos e de 10 a 14 anos nas regiões Norte e Sul, que apresentaram taxas maiores do que as observadas na faixa de 15 a 19 anos (Tabela 2).

**Tabela 2:** Taxas específicas por idade atribuídas à doença falciforme em crianças e adolescentes, por biênios, iniciados em 2000–2001 e finalizados em 2018–2019, no Brasil.

Localidade/biênio	Taxas^a^ em crianças		Taxas^a^ em adolescentes	
	0–4 anos	5–9 anos	10–14 anos	15–19 anos
Brasil				
2000–2001	0,20	0,11	0,08	0,13
2018–2019	0,32	0,16	0,09	0,28
Norte				
2000–2001	0,22	0,08	0,03	0,03
2018–2019	0,31	0,17	0,03	0,20
Nordeste				
2000–2001	0,23	0,08	0,11	0,16
2018–2019	0,45	0,17	0,16	0,51
Sudeste				
2000–2001	0,20	0,16	0,07	0,15
2018–2019	0,27	0,16	0,07	0,21
Sul				
2000–2001	0,08	0,02	0,06	0
2018–2019	0,05	0,05	0	0,07
Centro Oeste				
2000–2001	0,25	0,12	0,08	0,24
2018–2019	0,49	0,30	0,12	0,26

^a^ Taxas específicas por idade apresentadas por 100 mil pessoas-ano.

A visualização da disposição temporal dos coeficientes em gráficos permite a interpretação de maior magnitude dos óbitos nas faixas etárias de zero a quatro anos e de 15 a 19 anos, ao mesmo tempo em que sugere tendência de elevação das taxas ao longo do tempo, tanto no Brasil quanto nas macrorregiões (Figura 2).

**Figura 2:** Tendências das taxas de mortalidade por doença falciforme, específicas por idade em crianças e adolescentes, por 100 mil pessoas-ano, no Brasil (a) e nas macrorregiões geográficas (Norte (b), Nordeste (c), Sudeste (d), Sul (e), Centro-Oeste (f)), por biênios, iniciando em 2000–2001 e finalizando em 2018–2019.

## DISCUSSÃO

O estudo teve o mérito de analisar a doença falciforme, tema ainda pouco explorado cientificamente no Brasil, embora cause importante sofrimento aos pacientes e suas famílias. O enfoque em crianças e adolescentes revelou que as faixas etárias de zero a quatro anos e de 15 a 19 anos experimentaram as maiores taxas de mortalidade, tanto no Brasil como um todo quanto nas macrorregiões. As taxas de mortalidade ajustadas apontam para uma tendência de aumento desses óbitos ao longo dos anos no país e sugerem que, em média, o Brasil perde anualmente cerca de 125 crianças, devido às complicações de transtornos falciformes.

Foi possível verificar que crianças e adolescentes pardos e pretos são as principais vítimas fatais da DF nacional e regionalmente. Embora constitua uma característica comum da doença^14^, é preciso ter em mente as metas da Política Nacional de Saúde Integral da População Negra^15^, na qual se acentua a necessidade de priorizar ações que visem a redução das disparidades étnico-raciais nas condições de saúde, destacando a DF. No contexto étnico-racial, chama a atenção que, mesmo na região Sul, caracterizada pela dominância da população de cor branca, metade do contingente de óbitos foi registrada em pessoas brancas e a outra metade em pessoas negras. Levando em conta a miscigenação típica da população brasileira, a expectativa é que a distribuição proporcional seja, possivelmente, ainda maior entre afrodescendentes do que as apresentadas neste estudo.

Os dois estratos etários mais afetados (zero a quatro e 15 a 19 anos) também merecem reflexão, pois representam momentos da vida com significado, demandas e necessidades diferentes. Nos primeiros anos, a expectativa seria de maior usufruto da Política Nacional de Atenção Integral às Pessoas com Doença Falciforme e outras Hemoglobinopatias^16^, na qual se promove a garantia do seguimento das pessoas diagnosticadas pelo Programa Nacional de Triagem Neonatal, ofertando abordagens terapêuticas efetivas, medidas educacionais ampliadas aos familiares, aumento do acesso aos serviços de saúde especializados e, por consequência, redução da morbimortalidade nas crianças que nascem com defeitos falciformes^16^. Mas é justamente o estrato de zero a quatro anos que experimenta as maiores taxas de mortes indistintamente no Brasil e nas regiões geográficas.

Uma avaliação de causas de óbitos em pessoas com DF no Brasil, mostrou que 10,4% das mortes ocorreram em menores de cinco anos^17^. No presente estudo, o fato de o estrato 0–4 anos ter taxas mais elevadas e tendência de aumento sugere que os investimentos na DF feitos até então carecem de maior efetividade. É necessário identificar onde estão ocorrendo as falhas para propor medidas de aprimoramento que perpassem desde a adesão e/ou revisão dos protocolos até a ampliação do acesso dessas crianças aos serviços de saúde que possam fazer diferença nas condições de saúde, qualidade de vida e sobrevida com a doença.

A mortalidade da doença tem importância também sobre a faixa etária entre 15 e 19 anos, sendo o segundo estrato em número de mortes delineando um cenário de perdas entre aqueles que superaram longa trajetória de crises, adoecimentos e hospitalizações. Essa questão precisa ser investigada para que se possa indicar o que estaria acontecendo na adolescência tardia, causando aumento no número de mortes por DF como causa básica, um defeito genético que foi controlado durante os primeiros anos de vida. Elucidar essa problemática será essencial para readequar a atenção nessa fase da vida, a qual é impregnada de desafios e incertezas, agravados pela DF. Do lado prático, manter a adesão ao regime terapêutico é um grande desafio que deve ser trabalhado pelo profissional de saúde^18^. Do lado científico, falta suprir lacunas com produção de evidências de alto nível, esclarecendo, por exemplo, o impacto da mudança do local de atendimento, que passa a ser ofertado em ambientes destinados aos adultos^19^.

No Brasil, a taxa de óbitos na faixa < 5 anos apresentou um aumento de 60% comparando-se os biênios 2000–2001 e 2018–2019. Um estudo restrito à população negra nos Estados Unidos apontou para o declínio de óbitos na mesma faixa etária com taxas caindo de 2,05 (1979–1989) para 0,47 (2015–2017), por 100 mil pessoas-ano^20^. Na França, a investigação da causa de óbito em crianças diagnosticadas com DF ao nascimento foi feita em um estudo^21^ de seguimento de 15 anos, encerrado em 2015. Os autores mostraram que as complicações envolvendo infecção e anemia foram as causas mais frequentes e mais da metade dos óbitos compartilhavam as duas condições.

A DF vem ganhando atenção das autoridades brasileiras desde o início do século XXI, mesmo que lentamente, com a criação do Programa Nacional de Triagem Neonatal^6^, no qual foram definidas algumas responsabilidades e atribuições. A Política Nacional de Atenção Integral às Pessoas com Doença Falciforme e outras Hemoglobinopatias foi lançada em 2005 e deu ênfase à abordagem do problema no Sistema Único de Saúde^16^. Medidas gerais foram tratadas no Manual de Educação em Saúde do Ministério da Saúde^18^. Em 2009, a doença foi listada entre as prioridades elencadas pela Política Nacional de Saúde Integral da População Negra^15^. Um documento de protocolo clínico e diretrizes terapêuticas foi elaborado em 2010, no qual se avaliou o uso da hidroxiuréia^22^. A decisão de incorporar a hidroxiuréia com dose terapêutica adequada às crianças de dois anos ou mais foi publicada em 2013^23^. As condutas básicas para tratamento de DF, lançadas em 2012, dão ênfase à propriedade das medidas profiláticas, principalmente nas crianças^5^. Apesar de todo esse arcabouço de políticas públicas, a mortalidade em crianças por DF ainda é crescente no país. Infecções, síndrome torácica aguda e sequestração esplênica aguda são condições evitáveis, mas comumente reportadas^8^, demonstrando que ainda é preciso ter atenção para descobrir as falhas e consertá-las.

A DF penaliza os pacientes duplamente, tendo em vista tanto a evitabilidade das manifestações clínicas quanto a baixa visibilidade que ela experimenta no Brasil, ainda que constitua um problema de saúde pública nos países em desenvolvimento^24^. Nesse sentido, o estudo teve o mérito de abordar os indicadores de mortalidade em âmbito nacional, contudo, algumas limitações devem ser enumeradas. Primeiramente, considerando a importância dos estudos epidemiológicos e sua contribuição para as políticas de saúde que visam a redução das desigualdades raciais em saúde, uma limitação foi não estimar taxas segundo cor da pele, devido à indisponibilidade de dados populacionais estratificados de acordo com tal característica para cada faixa etária e marco temporal. No entanto, apresentamos uma síntese da distribuição demográfica, o que reforçou a importância do problema no grupo populacional pardo e preto, regional ou nacionalmente.

Outra limitação foi o enfoque nos óbitos codificados como D57, não abrangendo causas básicas atribuídas a outras hemoglobinopatias (D56). Embora a possibilidade de erro na classificação dos óbitos e de subestimação das taxas não devam ser ignoradas, nossos resultados são próximos daqueles reportados para crianças no estado do Maranhão^25^.

## CONCLUSÃO

O estudo mostrou tendência de aumento dos óbitos atribuídos aos transtornos falciformes em crianças e adolescentes. Considerando que a magnitude dos óbitos foi mais evidente nos primeiros anos (0–4) e no final da adolescência (15–19), o estudo sugere que abordagens específicas por idade podem impactar no controle dos desfechos fatais causados pela DF no Brasil.
